# A novel nomogram model based on Ki-67 characteristic expression to predict prognosis in head and neck squamous cell carcinoma

**DOI:** 10.3389/fonc.2024.1376498

**Published:** 2024-04-08

**Authors:** Tianyi Wang, Lili Xue, Zhixin Li, Zhicong Hong, Niting Hu, Yi Li, Bing Yan

**Affiliations:** ^1^ State Key Laboratory of Oral Diseases & National Center for Stomatology & National Clinical Research Center for Oral Diseases & Department of Head and Neck Oncology Surgery, West China Hospital of Stomatology, Sichuan University, Chengdu, Sichuan, China; ^2^ Department of Stomatology, The First Affiliated Hospital of Xiamen University, Xiamen, Fujian, China; ^3^ Department of Otolaryngology Head and Neck Surgery, The First Affiliated Hospital of Xiamen University, Xiamen, Fujian, China

**Keywords:** Ki-67, head and neck squamous cell carcinoma, prognosis, survival, nomogram, prognostic prediction model

## Abstract

**Objectives:**

This study aimed to examine Ki-67’s correlation with clinicopathological characteristics of head and neck squamous cell carcinoma (HNSCC), evaluate its prognostic significance, and develop a Ki-67 integrated prognostic model.

**Methods:**

The retrospective study included 764 HNSCC patients hospitalized from 2012 to 2022. Data were sourced from medical records and immunohistochemical analysis of surgical specimens.

**Results:**

Ki-67 expression was significantly associated with sex, pathological grade, clinical stage, and metastasis, but not with age or recurrence. Higher Ki-67 levels were linked to poorer prognosis, as indicated by Kaplan-Meier survival analysis. Utilizing a Cox proportional hazards model, four prognostic factors were identified: age, recurrence, metastasis, and Ki-67 expression. These factors were used to construct a prognostic model and a nomogram. The model’s predictive accuracy was confirmed by a high concordance index and a reliable calibration curve.

**Conclusion:**

Ki-67 expression in HNSCC patients correlates with several clinicopathological features and serves as a negative prognostic marker. A prognostic model incorporating Ki-67 was successfully developed, offering a new tool for patient prognosis assessment in HNSCC.

## 1 Introduction

Head and neck cancer, comprising malignancies in the oral cavity, pharynx, larynx, thyroid gland, salivary glands, and other head and neck regions, is a prevalent malignancy worldwide. In 2020, global incidence rates reported approximately 377,713 new cases of oral cancer, 98,412 of oropharyngeal cancer, 84,254 of hypopharyngeal cancer, and 184,615 of laryngeal cancer (1). Squamous cell carcinoma (SCC), the predominant form of head and neck cancer, originates from the malignant transformation of the squamous epithelium in these regions and constitutes about 90% of all such cancers (2). Common primary sites include the oral cavity, pharynx, and larynx (3). Head and neck squamous cell carcinoma (HNSCC) is notably characterized by invasive growth and a propensity for lymph node metastasis, posing significant diagnostic and treatment challenges globally.

In this context, substantial research has been dedicated to identifying biomarkers for HNSCC (4–6), aiming to enhance disease management, particularly in diagnosis and prognosis. However, limitations in biomarker verification, insufficient quantity or quality of marker molecules, and detection challenges persist. Moreover, methodological flaws in some studies, such as inadequate sample sizes, further complicate these challenges (7–9). Currently, there is no universally accepted definitive prognostic or predictive biomarker for HNSCC.

Despite the heterogeneity observed in tumors, a common characteristic is their uncontrolled proliferative capacity (10). Ki-67, a prominent proliferation marker in human tumor cells, is crucial during interphase and mitosis, with its cellular distribution varying significantly throughout the cell cycle (11). It has been identified as an independent marker for predicting the prognosis of various malignancies, including those of the lung, breast, kidney, and prostate (12–15). In HNSCC, substantial evidence supports Ki-67’s prognostic value (16–19). Nonetheless, the validity of this evidence is often questioned due to small sample sizes, and conflicting views exist regarding Ki-67’s correlation with HNSCC prognosis (20, 21). Furthermore, the nomogram model based on Ki-67 characteristic expression for head and neck squamous carcinoma is currently not a focal point of research.

To address these discrepancies, this study endeavors to evaluate the prognostic value of Ki-67 and construct a prognostic model incorporating Ki-67 in HNSCC by analyzing a large patient cohort, thus offering more substantial and reliable evidence.

## 2 Materials and methods

This investigation encompassed a retrospective cohort of 764 patients diagnosed with four principal subtypes of head and neck squamous cell carcinoma (HNSCC): oral squamous cell carcinoma (OSCC), oropharyngeal squamous cell carcinoma (OPSCC), hypopharyngeal squamous cell carcinoma (HPSCC), and laryngeal squamous cell carcinoma (LSCC). These patients were admitted to our institution between 2012 and 2022. A thorough analysis of their medical records yielded vital clinicopathological data, including age, sex, primary tumor site, pathological grade, and clinical TNM stage, adjudicated per the 8th edition of the Union for International Cancer Control’s Cancer Staging Manual. Additionally, information on recurrence, metastasis, survival status, and duration of survival as of December 31, 2022, was meticulously collected.

To augment this data, immunohistochemical staining was performed on formalin-fixed, paraffin-embedded surgical specimens, primarily focusing on the expression of Ki-67. This study received full approval from our Institutional Review Board (Ethics approval number: WCHSIRB-D-2023-437) and was rigorously conducted in alignment with the ethical guidelines set forth in the Declaration of Helsinki.

### 2.1 Immunohistochemical staining

For immunohistochemical staining, 2μm thick sections were prepared from paraffin-embedded tissue blocks. The initial stage involved dewaxing and rehydrating these sections through sequential immersion in xylene, followed by ethanol solutions at concentrations of 100%, 85%, and 75%. Subsequently, they were rinsed in distilled water and immersed in citrate buffer. Antigen retrieval was facilitated by heating the sections in a microwave, comprising a 10-minute cycle at high power, an 8-minute interval, and a further 10-minute cycle at medium power. This process is crucial for exposing antigens to enhance antibody binding efficiency.

To quench endogenous peroxidase activity, the sections were treated with a 3% hydrogen peroxide solution for 10 minutes at ambient temperature. This was followed by the application of normal goat serum (Product code: AR1009, Boster Biological Technology, diluted 1:9) for 20 minutes to reduce non-specific binding. The sections were then incubated with the primary antibody (Product code: GB121141-100, Wuhan Servicebio Technology, diluted 1:100) overnight at 4°C. The following day, a secondary antibody (Product code: SP9002, Beijing Zhong Shan -Golden Bridge Biological Technology) was applied for 30 minutes at 37°C. Subsequent development with a diaminobenzidine (DAB) solution imparted a brownish-yellow coloration to the cell nuclei. Finally, the sections were washed, counterstained with hematoxylin for three minutes, rinsed, and mounted using gum.

### 2.2 Recognition and counting of Ki-67

The DAB staining methodology is employed to discern Ki-67-positive cells, characterized by the distinctive brownish-yellow coloration of their nuclei. A cell is classified as Ki-67 positive when its nucleus exhibits this specific hue, irrespective of the intensity of the staining. Regions manifesting the highest expression of Ki-67 are subjected to detailed examination under high magnification. This process involves quantifying the total number of Ki-67 positive cells and comparing it with the aggregate count of tumor cells present in the sample. The resulting ratio is subsequently converted into a percentage, representing the level of Ki-67 expression.

### 2.3 Statistical analysis

In this study, the expression of Ki-67 was evaluated through various categorical variables such as age, sex, pathological grade, clinical TNM stage, recurrence, and metastasis. Owing to the non-Gaussian distribution of Ki-67 expression within these groups, nonparametric statistical methods were employed for analysis. The Wilcoxon rank sum test facilitated the comparison of Ki-67 expression between two distinct groups, while the Kruskal-Wallis H test was used to evaluate differences among three or more groups. Specific group comparisons were conducted using Dunn’s multiple comparison test for specific group assessments.

To categorize patients into high and low Ki-67 expression cohorts, the optimal cut-off value derived from the receiver operating characteristic (ROC) curve and the median value of Ki-67 expression were utilized. Kaplan-Meier survival curves, coupled with log-rank tests, were applied to discern the disparities in overall survival between these groups. A Cox proportional hazards model was formulated, excluding pathological grade and clinical TNM stage as mediating variables. This model incorporated five key variables: Ki-67 expression, age, sex, recurrence, and metastasis. Nomograms were constructed from significant predictors identified in the Cox regression analysis to prognosticate patient outcomes. The model’s accuracy was quantified using the concordance index (C-index) and further validated through Bootstrap resampling techniques.

Considering the heterogeneity inherent in head and neck tumors, separate Cox models were developed for different anatomical regions.

For statistical analysis and graphic visualization, R software version 4.3.2 (R Foundation for Statistical Computing, Vienna, Austria) and GraphPad Prism version 10.0.2 (GraphPad Software, San Diego, California, USA) were employed. A threshold of p < 0.05 was set to determine statistical significance.

## 3 Results

### 3.1 General information

This study encompassed a cohort of 764 patients. Among these, 372 individuals (48.7%) were aged 60 or younger, while 392 (51.3%) were older than 60. The distribution by sex was notably skewed, with 596 patients (78.0%) being male and 168 (22.0%) females. Pathologically, the majority, 529 patients (69.2%), were diagnosed with moderately differentiated squamous cell carcinoma (MDSCC). This was followed by 109 (14.3%) with well-differentiated squamous cell carcinoma (WDSCC) and 126 (16.5%) with poorly differentiated squamous cell carcinoma (PDSCC). In terms of clinical staging, 433 patients (56.7%) presented with advanced stage disease (Stage III-IV), while 331 (43.3%) were at an early stage (Stage I-II). A significant majority, 683 patients (89.4%), exhibited no recurrence, in stark contrast to the 81 patients (10.6%) who experienced a recurrence. Neck or distant metastases occurred in 480 (62.8%) patients, while 284 (37.2%) did not have any. As for Ki-67 expression, the median value was 50%, with an interquartile range of 40% and an average of 48.787 ± 22.165%. Regarding tumor location, 443 cases (58.0%) of HNSCC occurred in the oral cavity, 26 (3.4%) in the oropharynx, 76 (9.9%) in the hypopharynx, and 219 (28.7%) in the larynx (**Table 1**).

**Table 1:** General clinicopathological information of 764 cases.

Clinical characteristics	Cases, n (%)
All Patients	764(100)
Age(year)	
≤60	372(48.7)
>60	392(51.3)
Sex	
Male	596(78.0)
Female	168(22.0)
Grade	
WDSCC	109(14.3)
MDSCC	529(69.2)
PDSCC	126(16.5)
Clinical Stage	
Early Stage (I-II)	331(43.3)
Advanced Stage (III-IV)	433(56.7)
Recurrence	
Non-rec	683(89.4)
Recurrent	81(10.6)
Metastasis	
Non-meta	480(62.8)
Metastatic	284(37.2)
Ki-67(%)	
Median [IQR]	50[30-70]
Mean ± SD	48.787 ± 22.165
Site	
Oral cavity	443(58.0)
Oropharynx	26(3.4)
Hypopharynx	76(9.9)
Larynx	219(28.7)

### 3.2 Correlation of Ki-67 expression with clinicopathological features

In this study, no statistically significant variance was observed in the mean Ki-67 expression when comparing patients aged 60 or younger with those older than 60, as well as between patients with local recurrence and those without. However, a notable difference in Ki-67 mean expression was evident between sexes, with males exhibiting higher levels than females. Additionally, a clear trend was observed in Ki-67 expression relative to pathological grade; as the grade worsened, Ki-67 expression notably increased. This suggests that tumor cells with poorer differentiation are associated with elevated Ki-67 expression.

Moreover, a significant variation in Ki-67 expression was identified between different clinical TNM stages. Patients in the early stages exhibited lower Ki-67 expression compared to those in advanced stages. This difference was statistically significant, highlighting the potential of Ki-67 as a marker for disease progression.

**Figure 1:**
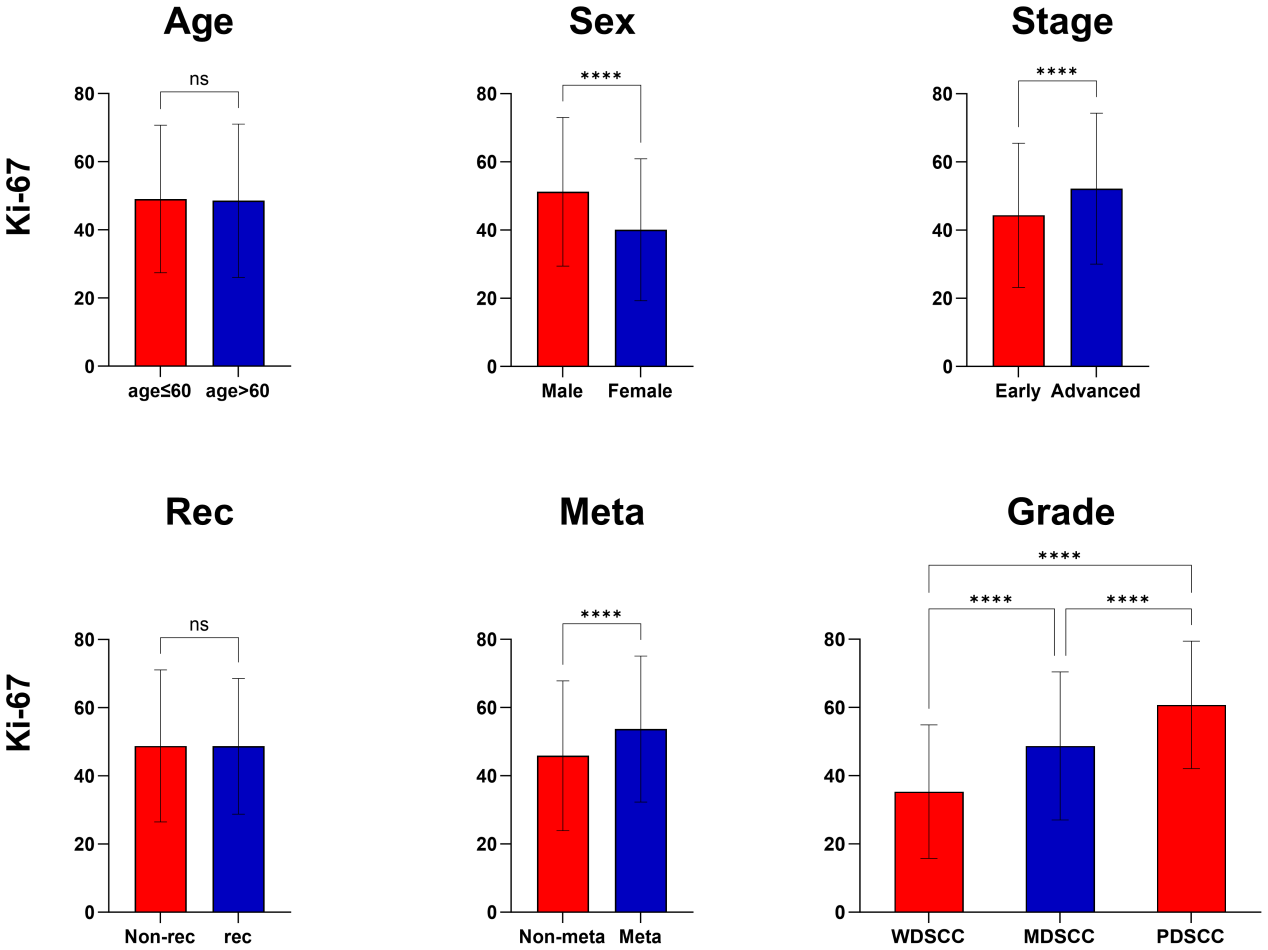
Correlation of Ki-67 expression with clinicopathological features. ****, P<0.0001, ns, no significance.

Furthermore, there was a demonstrable correlation between Ki-67 expression and the occurrence of metastasis. Ki-67 levels were significantly higher in patients who had lymph node or distant metastasis compared to those without such metastatic spread (**Table 2**) (**Figure 1**).

**Table 2:** Correlation of Ki-67 expression with clinicopathological features.

Variable	n	Ki-67 Expression		P-value
		Mean	SD	
Age				
≤60 years	372	49.048	21.621	0.716
>60 years	392	48.538	22.496	
Sex				
Male	596	51.238	21.798	<0.0001
Female	198	40.089	20.817	
Grade				
WDSCC (A)	109	35.330	19.589	A<B <0.0001
MDSCC (B)	529	48.709	21.696	B<C <0.0001
PDSCC (C)	126	60.754	18.677	A<C <0.0001
Clinical Stage				
Early (I-II)	331	44.353	21.169	<0.0001
Advanced (III-IV)	433	52.176	22.153	
Recurrence				
Non-rec	683	48.799	22.319	0.9566
Recurrent	81	48.679	19.880	
Metastasis				
Non-meta	480	45.869	21.942	<0.0001
Metastatic	284	53.718	21.412	

### 3.3 Correlation of Ki-67 expression with patient prognosis

**Figure 2:**
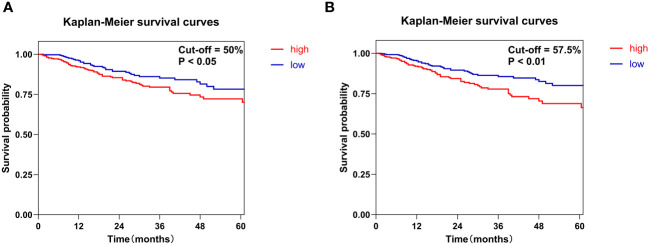
Kaplan-Meier survival curves were constructed to evaluate the overall survival of patients with HNSCC based on Ki-67 expression levels. Specifically, Ki-67 expression was classified into high and low groups using two different methods. **(A)** The median value of Ki-67 expression of 50% served as the cut-off. **(B)** The cut-off was set at the optimal threshold of 57.5%, which was determined from the ROC curve.

This study employed two distinct cut-off values to optimize the grouping of Ki-67 expression, thereby enabling a more precise evaluation of its expression levels. The first method involved dividing the sample into two groups using the median value of 50% as the cut-off. An alternative approach determined the optimal cut-off value via the receiver operating characteristic (ROC) curve, which was identified as 57.5%. This cut-off value demonstrated a specificity of 0.589, a sensitivity of 0.579, and an area under the curve (AUC) of 0.605. Kaplan-Meier survival curves and log-rank test results indicated that the prognosis for patients in the high Ki-67 expression group was significantly poorer compared to those in the low expression group (P<0.05). Notably, the cut-off value derived from the ROC curve proved more effective in stratifying Ki-67 expression than the median-based approach (**Figure 2**).

Multivariate Cox regression analysis of HNSCC identified age, recurrence, metastasis, and Ki-67 expression as independent prognostic risk factors (all hazard ratios >1, all P<0.05) (**Table 3**). Patients with high Ki-67 expression, advanced age, presence of metastasis, and recurrence had a poorer prognosis than those with lower Ki-67 levels, younger age, absence of metastasis, and no recurrence. Sex did not show a significant impact on prognosis (P>0.05).

Furthermore, separate analyses of Cox proportional hazards models for OSCC, OPSCC, HPSCC, and LSCC revealed that Ki-67 expression was an independent prognostic factor solely in OSCC. In contrast, its prognostic relevance was not significant in OPSCC, HPSCC, and LSCC. (Due to the limited sample size, the HRs for Sex and Recurrence in HPSCC were calculated to be 0.000, lacking a 95% confidence interval (CI). Similarly, in LSCC, the HRs for sex were also found to be 0.000 without a 95% CI. Consequently, we opted to exclude these variables from the separate Cox regression models) (**Table 3**).

**Table 3:** Multivariate analysis of overall survival by Cox proportional hazards model in the whole head and neck region and in sub-sites.

Characteristic	Cox proportional hazards regression	
	HR (95%CI)	P value
Head and Neck		
Age	1.027(1.009-1.045)	0.003
Sex		
Male		
Female	1.065(0.652-1.740)	0.802
Recurrence		
Non-rec		
Recurrent	1.865(1.147-3.031)	0.011
Metastasis		
Non-meta		
Metastatic	3.161(2.147-4.655)	0.000
Ki-67	1.009(1.004-1.018)	0.040
Oral Cavity		
Age	1.043(1.014-1.072)	0.003
Sex		
Male		
Female	1.317(0.718-2.416)	0.374
Recurrence		
Non-rec		
Recurrent	4.316(2.327-8.005)	0.000
Metastasis		
Non-meta		
Metastatic	4.874(2.564-9.266)	0.000
Ki-67	1.014(1.0001-1.027)	0.049
Oropharynx		
Age	1.029(0.975-1.086)	0.307
Sex		
Male		
Female	3.270(0.639-16.738)	0.155
Recurrence		
Non-rec		
Recurrent	1.391(0.267-7.257)	0.695
Metastasis		
Non-meta		
Metastatic	2.481(0.734-8.391)	0.144
Ki-67	0.979(0.954-1.005)	0.111
Hypopharynx		
Age	1.043(0.999-1.090)	0.056
Metastasis		
Non-meta		
Metastatic	1.569(0.581-4.238)	0.375
Ki-67	0.981(0.958-1.005)	0.119
Larynx		
Age	1.034(0.996-1.073)	0.077
Recurrence		
Non-rec		
Recurrent	0.561(0.074-4.276)	0.577
Metastasis		
Non-meta		
Metastatic	2.195(0.870-5.536)	0.096
Ki-67	1.016(0.994-1.038)	0.162

**Figure 3:**
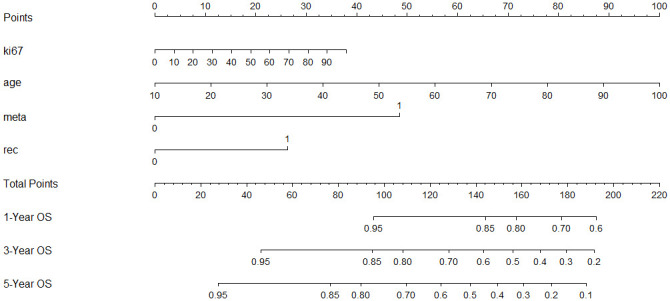
A constructed nomogram for prognostic prediction of a patient with HNSCC. The total points were calculated by summing up the points related to the patient’s age, Ki-67 expression, recurrence, and metastasis. The patients’ 1-, 3-, and 5-year predicted survival rates could be obtained using the total points.

**Figure 4:**
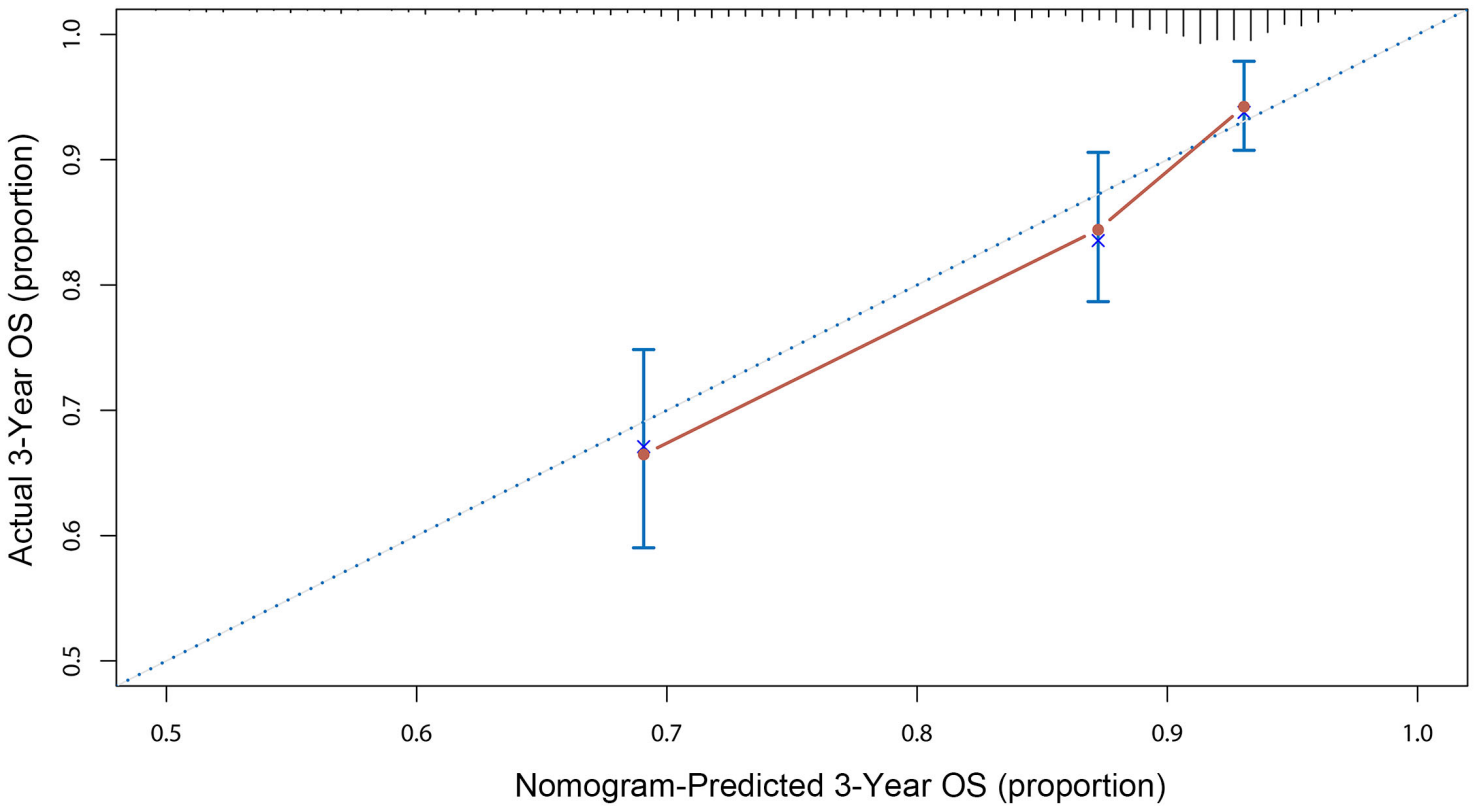
Calibration curve of 3-Yeas OS for HNSCC patients in the training cohort.

Using significant predictors from the Cox model, a nomogram was constructed (**Figure 3**), enabling the prediction of 1-, 3-, and 5-year survival rates based on the total points accumulated from a patient’s age, Ki-67 expression, recurrence status, and metastasis presence. For example, a 60-year-old patient with cervical lymph node metastasis, no recurrence, and a Ki-67 expression of 70% would score a total of 130 points, correlating to predicted survival rates of 87.5% at 1 year, 68% at 3 years, and 55% at 5 years. The nomogram’s C-index was 0.721, indicating good predictive accuracy for overall survival, with the calibration curve showing close alignment between predicted and observed 3-year survival rates (**Figure 4**).

**Figure 5:**
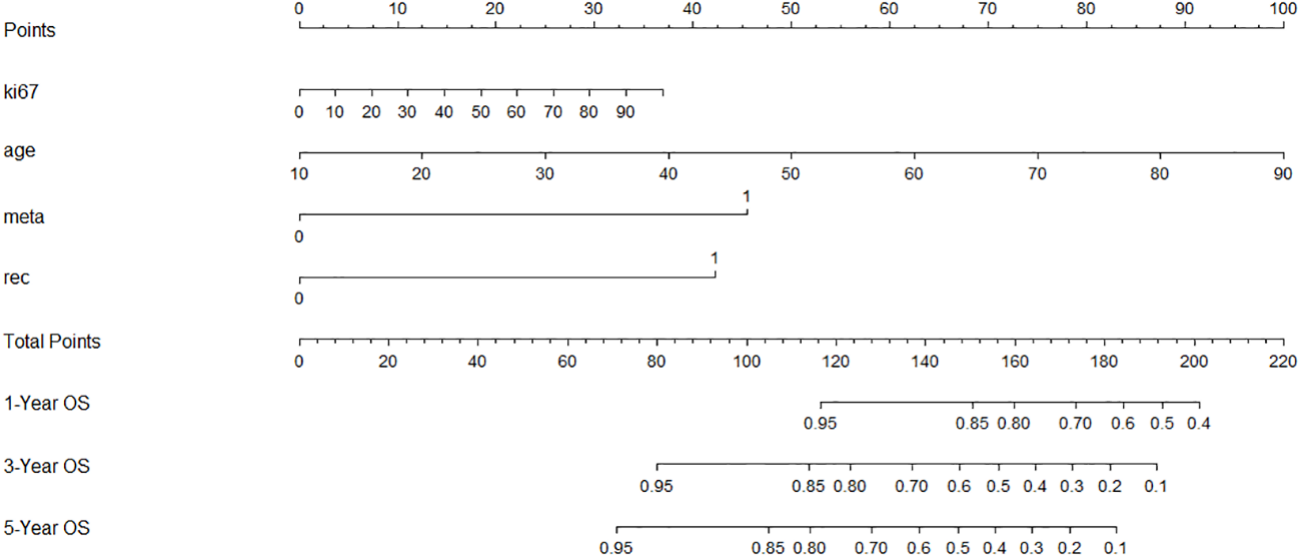
A constructed nomogram for prognostic prediction of a patient with OSCC.

The site-specific Cox proportional hazards model revealed an adverse effect of Ki-67 expression on the prognosis of OSCC. In addition, the overall cohort contained a large proportion of OSCC patients. Therefore, based on the nomogram for head and neck squamous carcinoma, a separate nomogram for OSCC was constructed (**Figure 5**).

## 4 Discussion

The identification of prognostic biomarkers and the development of prognostic models for head and neck squamous cell carcinoma (HNSCC) are pivotal for predicting patient outcomes and guiding clinical decision-making. This facilitates the adoption of personalized clinical strategies (22). Ki-67, a nuclear protein initially discovered in proliferating Hodgkin’s lymphoma cells by Gerdes et al. (23), is exclusively expressed during mitosis and absent in quiescent cells. Its expression, regulated by cell cycle-dependent transcription and protein degradation (24), marks it as a critical cell proliferation marker. Despite its established prognostic efficacy in various tumors, insights into Ki-67’s role in HNSCC remain varied, influenced by factors like sample size, detection techniques, and cut-off values for grouping (25–27).

In our study, Kaplan-Meier curves and log-rank tests identified Ki-67 as a prognostic risk factor for HNSCC. Cox multivariate analysis, controlling for confounders, confirmed Ki-67’s role as an independent prognostic factor alongside age, recurrence, and metastasis. Our study’s prognostic model, incorporating Ki-67, age, recurrence, and metastasis, revealed that recurrence and metastasis had a more substantial impact on prognosis than Ki-67 expression and age, as evidenced by their higher hazard ratio values. The model’s C-index was 0.721, indicating excellent predictive efficacy (28). The calibration curve corroborated the nomogram’s accuracy in reflecting actual patient survival.

Furthermore, within the Cox proportional hazards model, while the HR for Ki-67 expression exceeded 1, it was lower than that of other variables. This can be attributed to the Ki-67 expression being analyzed as a continuous variable with values ranging from 0 to 100. The HR for Ki-67 expression in the overall cohort was 1.009, representing that if the expression of Ki-67 increased by 1, the risk of patient death increased by 1.009. Upon further analysis, by stratifying Ki-67 expression into two groups based on a predefined cut-off value for the Cox model, we observed a substantially increased HR for Ki-67 expression. Nevertheless, we advocate for the utilization of Ki-67 expression as a continuous variable. This approach not only preserves the integrity of the data but also ensures that the Nomogram remains uninfluenced by arbitrary cut-off values for Ki-67 expression, thereby enhancing its utility.

Unlike prior studies that often relied solely on clinical, gene transcription, or gene expression data for prognostic modeling, our research uniquely integrated clinical data with immunohistochemical staining. Immunohistochemical staining, a standard procedure in tumor pathology, is both cost-effective and time-efficient. It proves more manageable in clinical settings than gene expression analysis. By simply assessing Ki-67 levels alongside key clinicopathological features, one can predict a patient’s prognosis using the nomogram. This predictive insight equips clinicians with solid evidence, facilitating informed decision-making about subsequent treatment steps. For instance, a poor predicated prognosis may prompt the consideration of active measures, including postoperative radiotherapy and chemotherapy. This has a positive effect on the overall prognosis of the patient. Moreover, the prognostic model’s presentation through a nomogram offers clarity, ease of understanding, and practical applicability.

Our research also explored Ki-67 expression’s correlation with clinicopathological features in HNSCC patients. Elevated Ki-67 expression in advanced-stage and poorly differentiated tumors highlighted its link to cell proliferation (29). Notably, Ki-67 expression was significantly higher in males than females, possibly reflecting variations in sex hormones or lifestyle factors like tobacco and alcohol use (30, 31). HPV infection status also appeared to influence proliferative activity (32). A significant difference in Ki-67 expression across different sites necessitated subgroup analysis, revealing Ki-67 as a prognostic biomarker only in OSCC. Because of this, we additionally constructed a separate nomogram for OSCC, demonstrating a prognostic model for containing Ki-67 expression. The prognostic significance of Ki-67 expression in OPSCC, HPSCC, and LSCC was not established, highlighting the need for further research, particularly due to sample size constraints.

Previous studies on Ki-67 expression in HNSCC have employed various cut-off values, including ROC curve-derived values (33), medians (13), quartiles (34), and data distribution-based values (17). Our study utilized both the median and ROC-derived optimal cut-off values, finding the latter to offer more significant prognostic variance. In calculating the cut-off value via the ROC curve, we analyzed a substantial cohort to establish this threshold. In future research, it is imperative to undertake prospective studies to affirm the prognostic significance of Ki-67 expression and to address pivotal concerns, including the determination of cut-off values. Various methodologies for quantifying Ki-67 expression, such as positive cell and area ratios, underscore the need for standardized assessment procedures. The lack of standardization, as highlighted in breast cancer studies (35, 36), calls for the establishment of consistent protocols for Ki-67 assessment in HNSCC.

In summary, this research encompassed a comprehensive analysis of 764 patients hospitalized from 2012 to 2022. It meticulously investigated the correlation between Ki-67 expression and a spectrum of clinicopathological characteristics in HNSCC. Our survival analysis unequivocally established Ki-67 as a negative prognostic biomarker for HNSCC. We developed a nomogram as a prognostic prediction model, integrating Ki-67 expression, which promises to contribute significantly to future clinical practice. However, it is crucial to acknowledge the inherent heterogeneity of head and neck tumors, underscoring the need for further validation of our findings in specific sites such as the oropharynx, hypopharynx, and larynx. Future research directions should focus on refining methodologies, including Ki-67 counting techniques, antigen retrieval processes, and determining the optimal cut-off value for Ki-67 expression. The ultimate goal is to establish a standardized and universally applicable procedure for assessing Ki-67 in HNSCC, thereby enhancing the accuracy and reliability of prognostic predictions in clinical settings.

## Data availability statement

The raw data supporting the conclusions of this article will be made available by the authors, without undue reservation.

## Ethics statement

The studies involving humans were approved by West China Hospital of Stomatology Institutional Review Board. The studies were conducted in accordance with the local legislation and institutional requirements. The participants provided their written informed consent to participate in this study.

## Author contributions

TW: Conceptualization, Data curation, Formal analysis, Investigation, Methodology, Visualization, Writing – original draft, Writing – review & editing. LX: Data curation, Formal analysis, Investigation, Visualization, Writing – review & editing. ZL: Data curation, Formal analysis, Investigation, Writing – review & editing. ZH: Data curation, Investigation, Writing – review & editing. NH: Data curation, Investigation, Writing – review & editing. YL: Methodology, Supervision, Validation, Writing – review & editing. BY: Conceptualization, Formal analysis, Investigation, Methodology, Project administration, Supervision, Writing – review & editing.
